# Reliability of CKD-EPI predictive equation in estimating chronic kidney disease prevalence in the Croatian endemic nephropathy area

**DOI:** 10.11613/BM.2018.010701

**Published:** 2017-11-24

**Authors:** Mirjana Fuček, Živka Dika, Sandra Karanović, Ivana Vuković Brinar, Vedran Premužić, Jelena Kos, Ante Cvitković, Maja Mišić, Josip Samardžić, Dunja Rogić, Bojan Jelaković

**Affiliations:** 1 Department of Laboratory Diagnostics, University Hospital Center Zagreb, School of Medicine University of Zagreb, Zagreb, Croatia; 2 Department of Nephrology, Hypertension and Dialysis, University Hospital Center Zagreb, School of Medicine University of Zagreb, Zagreb, Croatia; 3 Institute for Public Health, Brodsko Posavska County, Slavonski Brod, Croatia; 4 Department of Pathology, General Hospital “Dr. Josip Benčević“, Slavonski Brod, Croatia

**Keywords:** chronic kidney disease, estimated glomerular filtration rate, predictive equation, endemic nephropathy

## Abstract

**Introduction:**

Chronic kidney disease (CKD) is a significant public health problem and it is not possible to precisely predict its progression to terminal renal failure. According to current guidelines, CKD stages are classified based on the estimated glomerular filtration rate (eGFR) and albuminuria. Aims of this study were to determine the reliability of predictive equation in estimation of CKD prevalence in Croatian areas with endemic nephropathy (EN), compare the results with non-endemic areas, and to determine if the prevalence of CKD stages 3-5 was increased in subjects with EN.

**Materials and methods:**

A total of 1573 inhabitants of the Croatian Posavina rural area from 6 endemic and 3 non-endemic villages were enrolled. Participants were classified according to the modified criteria of the World Health Organization for EN. Estimated GFR was calculated using Chronic Kidney Disease Epidemiology Collaboration equation (CKD-EPI).

**Results:**

The results showed a very high CKD prevalence in the Croatian rural area (19%). CKD prevalence was significantly higher in EN then in non EN villages with the lowest eGFR value in diseased subgroup.

**Conclusions:**

eGFR correlated significantly with the diagnosis of EN. Kidney function assessment using CKD-EPI predictive equation proved to be a good marker in differentiating the study subgroups, remained as one of the diagnostic criteria for EN.

## Introduction

Over the past three decades the prevalence of chronic kidney disease (CKD) is on the rise worldwide (*1*). The Kidney Disease Outcomes Quality Initiative (KDOQI) consequently published guidelines for diagnosis of CKD and postulated the five stages for disease classification (CKD_1-5_), based on disease severity. Glomerular filtration rate (GFR) was set as the most important criteria for disease severity determination according to the 2002 guidelines (*2*).

In order to better assess disease progression, in 2012 the Kidney Disease: Improving Global Outcomes (KDIGO) group revised the CKD staging system and issued the new KDIGO CKD guidelines (*3*). This new disease staging system was based on five eGFR stages (the third stage (G3) was furthermore stratified into substages G3a and G3b) and three stages of albuminuria determined on the basis of the albumin-to-creatinine ratio (ACR). However the definition of CKD did not change, *i.e.* CKD was defined as an eGFR < 60 mL/min/1.73m^2^ during the period of 3 months (*4*). Based on these indicators and following the results of a meta-analysis that confirmed the independent value of estimated GFR (eGFR) and albuminuria for predicting mortality due to cardiovascular events, acute kidney injury, progressive CKD and end-stage renal disease (ESRD), a “heat map” was generated according to which patients could be classified into four risk categories (depending on renal and cardiovascular outcomes): low (stages G1-A1, G2-A1), moderate (stages G1-A2, G2-A2, G3a-A1), high (G1-A3, G2-A3, G3a-A2, G3b-A1) and very high risk (G3a-A3, G3b-A2-3, all G4 and G5) (*5*).

**Figure 1:**
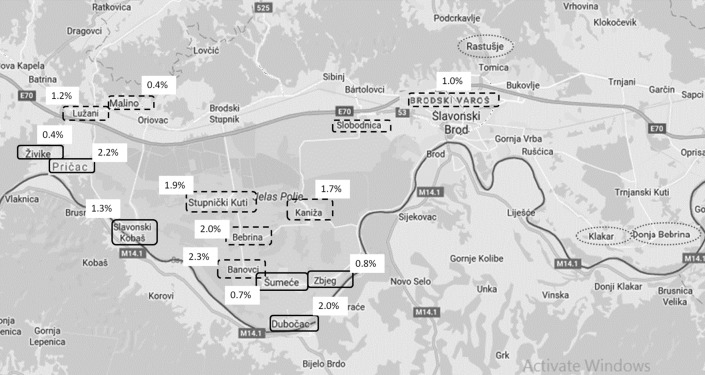
Endemic focus in Croatia with respective epidemiological data. The average prevalence of endemic nephropathy in endemic villages from 1980 to 1991 is presented as percentage (*20*). EN-villages enrolled in this study are rounded with a full black line; other EN villages (not included in this study) are rounded with dashed black line; non-EN villages are rounded with a dotted line.

Epidemiological data on the prevalence of CKD in Croatia are still missing despite the increasing awareness of public health problems associated with CKD. There is only one preliminary report on prevalence of CKD in Croatian rural population which showed that CKD is more frequently present in this area comparing to other regions (*6*). One specific cause of CKD in Croatia is endemic nephropathy (EN). It was reported in Posavina and comprises 14 villages situated west of the city of Slavonski Brod (Figure 1) with a population of 12,686 inhabitants according to the census from 2011 (*7*).

EN is a chronic tubulointerstitial nephropathy with subtle onset and characteristically gradual progression to ESRD with no significant gender difference - some reports in which EN is more frequent in women than in men are probably incorrect because before developing a kidney disease many men die of some cardiovascular disease (*8*).

The cause of EN is chronic aristolochic acid poisoning through contaminated food in genetically predisposed individuals (*9*). The adducts of aristolochic acid were isolated from the renal cortex of EN patients only, and not from patients with CKD of different etiology. Furthermore, a fingerprint mutation of the p53 gene (an AT:TA transversion) was confirmed in EN patients (*9*). Today, EN is considered as an environmental form of aristolochic acid nephropathy. Clinical course and disease progression of EN do not differ from other tubulointerstitial nephropathies (*10*). For now, there are no specific biomarkers for EN diagnosis and diagnostic criteria have not been precisely established. This means that various countries used different criteria. However, all these criteria have serious disadvantages (outdated, uneven and not in accordance with the new recommendations for the classification of renal diseases). Recently, a consensus document with new diagnostic criteria was accepted and it is hoped that researcher from all countries will start to use these compilation of criteria (*11*).

In EN, GFR is, just as in other CKD, considered a marker of overall renal function (*12*). Furthermore, individual patients’ risk estimation, prognosis of the disease clinical course and treatment decisions are based upon patients’ GFR. At the population level, it is important to accurately determine the prevalence of CKD to enable patient care planning and to estimate the need for renal replacement therapy as this is essential for planning public health budget, especially in areas with high incidence of CKD such as the Croatian areas with EN.

Because of higher exposure to aristolochic acid in the past, prevalence of CKD is higher in Croatian endemic area than in non-endemic villages. Our aims were to determine the prevalence of CKD using Chronic Kidney Disease Epidemiology Collaboration (CKD-EPI) equation and to analyse diagnostic value of eGFR in diagnosis and stratification of subjects from EN area. The results obtained contribute to the detection of differences in the prevalence of CKD stages and in their frequency in endemic and non-endemic villages.

## Materials and methods

### Subjects

This cross-sectional observational study is based on data collected by field research between 2008 and 2010 as a part of the Croatian Ministry of Science research project entitled “Endemic Nephropathy in Croatia: Epidemiology, Diagnosis, Etiology” (108-0000000329). The survey was approved by the Ethical Committee of the Croatian National Institute of Public Health, the School of Medicine University of Zagreb and General Hospital ‘Dr. Josip Benčević’ Slavonski Brod.

A total of 1573 adult inhabitants from six endemic villages (Pričac, Slavonski Kobaš, Živike, Šumeće, Zbjeg and Dobočac) and three non-endemic villages (Klakar, Donja Bebrina, Rastušje) were invited to participate on a door-to-door basis and were enrolled after signing informed consent. The study included significant and comparable percentages of participants from each village over previous researches (Table 1) (*13*). A physician, nurse and technician went to the village where an extensive epidemiological questionnaire was completed and clinical examination (including measurement of blood pressure, height, weight) and sampling for laboratory analyses of blood and urine were performed. Venous blood (4 mL in K_2_EDTA blood collection tube for red blood cell count and 8.5 mL in tube with Silica Clot Activator-SST for biochemistry tests; all BD Diagnostic, Sparks, USA) and 50 mL of second morning urine sample (Sterile Specimen Collection Cup of 120 mL without preservative; BD Diagnostic, Sparks, USA) were collected from each subject. Blood samples were centrifuged 10 minutes at 3500 rpm within 2 hours of collection. All samples were transported the same day (in conditions that ensure the stability of the analyte) to the Department of Laboratory Diagnostics, University Hospital Center Zagreb, and testing was performed immediately upon acceptance.

**Table 1:** The percentage of the population enrolled in the study

**Village**	**Adult population, N**	**Enrolled population, N (%)**
Pričac	76	51 (67)
SLavonski Kobaš	893	585 (66)
Živike	183	103 (56)
Šumeće	415	252 (61)
Zbjeg	336	129 (38)
Dubočac	163	106 (65)
Klakar	201	90 (45)
Donja Bebrina	317	153 (48)
Rastušje	219	104 (47)
Total	2803	1573 (56)

According to the modified World Health Organization (WHO) criteria, which have been used in Croatia for the last 40 years, participants in endemic villages were classified into diseased, suspected of having EN, those at risk of developing EN and others, farmers from villages who were completely unrelated to EN (*14*). Subjects from endemic villages with missing key data for classification were not included in the study. Inclusion criteria were based on the following data: (a) positive family history of EN and/or living > 20 years in EN village; (b) low molecular weight proteinuria (alpha1-microglobulin > 10 mg/L or alpha1-microglobulin/creatinine > 14 mg/g); (c) serum creatinine > 132.6 µmol/L; (d) anaemia (haemoglobin (Hb) < 120 g/L if male, Hb < 113 g/L if female), and (e) exclusion of other renal diseases (including diabetes).

Participants were considered “diseased” if they were positive for criteria listed as (a), (b), (c), (d) and (e); or (b), (c), (d) and (e); and/or (a), (b), (d) and (e). Patients with positive criteria listed as (a) and (b) or (b) and (d) were “suspected of having EN” and “at risk” if they were from a family with EN. To exclude other renal diseases, laboratory data and imagining techniques were used when necessary. The control group was comprised of subjects who did not have EN according to the previously listed criteria (subjects from non-endemic villages).

Due to the fact that diabetes and hypertension are major causes of CKD, participants were additionally divided into subjects with hypertension according to the European Society of Hypertension classification (*i.e.* systolic blood pressure of ≥ 140 mmHg and diastolic blood pressure of ≥ 90 mmHg, measured with an automated device (Omron HEM-907, Matsusaka, Japan); and/or taking any antihypertensive agent) and subjects with diabetes (DM) according to the American Diabetes Association guidelines (fasting blood glucose ≥ 7.0 mmol/L measured by enzymatic UV method with hexokinase and/or taking drugs to lower blood glucose) (*15*, *16*). Furthermore, participants were divided into three age groups: group I (age 18 - 42); group II (age 43 - 66), and group III (age ≥ 67).

### Methods

Blood and urine samples were analyzed using standard laboratory methods. Red blood cell count (RBC) was determined using laser light scattering technology on the Cell Dyn Sapphire (Abbott Diagnostic, Illinois, USA) and Sysmex XE 5000 (Sysmex Europe GmbH, Norderstedt, Germany) analyzers. Serum (SCr) and urine creatinine (UCr) were measured on the Olympus AU 2700 analyzer (Beckman Coulter, California, USA) using the Jaffé kinetic uncompensated method with continuous measurement. Calibration was performed using proprietary calibrators traceable to isotope dilution mass spectrometry (IDMS) method and Standard Reference Material (SRM) 909B of the National Institute of Standards and Technology (NIST, USA) for serum and the NIST reference material SRM 914a (substance creatinine purity of 99.7 ± 0.3%) for urine samples. Urine alpha1-microglobulin (alpha1-MG) was measured by immunonephelometric assay (on the BN II nephelometer, Siemens Healthcare Diagnostics Inc, Tarrytown, USA). Continuous internal quality control was performed throughout the study using quality control materials provided by the respective manufacturers. For CKD staging, urine albumin (U-Alb) was measured in the second morning sample by immunonephelometric assay (BN II nephelometer, Siemens Healthcare Diagnostics Inc, Tarrytown, USA) standardized using primary ERM-DA470 calibrators with a method sensitivity of 3.0 mg/L. The same tests were performed in the endemic and the control group.

Glomerular filtration rate (eGFR in mL/min/1.73m^2^) was calculated by applying SCr to CKD-EPI equation (*17*). The equations are presented below (Eq. 1 for women and Eq. 2 for men).

(Eq. 1)

if SCr ≤ 62 μmol/L GFR(mL/min/1.73m^2^) = 144 x (SCr (μmol/L) / 62) ^- 0.329^ x (0.993)^age (years)^

if SCr > 62 μmol/L GFR(mL/min/1.73m^2^) = 144 x (SCr (μmol/L) / 62) ^- 1.209^ x (0.993)^age (years)^

(Eq. 2)

if SCr ≤ 80 μmol/L GFR (mL/min/1.73m^2^) = 141 x (SCr (μmol/L)/80) ^- 0.411^ x (0.993)^age (years)^

if SCr > 80 μmol/L GFR (mL/min/1.73m^2^) = 141 x (SCr (μmol/L) / 80) ^– 1.209^ x (0.993)^age (years)^

Because the CKD-EPI equation was evaluated according to the SCr values measured by compensated Jaffé method, SCr values of all subjects were corrected before inclusion in the equation using the linear regression equation: y = 0.95 x + 0.44 (y = compensated Jaffè; x = uncompensated Jaffè) (*18*).

The revised KDIGO classification was used to define CKD stages which includes five stages of eGFR: G1 (≥ 90 mL/min/1.73m^2^), G2 (60 - 89 mL/min/1.73m^2^), G3a (45 - 59 mL/min/1.73m^2^), G3b (30 - 44 mL/min/1.73m^2^), G4 (15 - 29 mL/min/1.73m^2^), G5 (< 15 mL/min/1.73m^2^) and three levels of albuminuria based on the albumin-to-creatinine ratio (ACR): A1 (< 30 mg/g), A2 (30 – 300 mg/g) and A3 (> 300 mg/g).

CKD prevalence was assessed using the eGFR calculated with CKD-EPI equation. We evaluated the overall prevalence of CKD and the prevalence of stages 3-5 (CKD_3-5_) in all subgroups depending on gender, age and place of recruitment. CKD was defined as eGFR < 60mL/min/1.73m^2^.

### Statistical analysis

The SPSS software (SPSS Version 18, SPSS Inc., Chicago, USA) was used for statistical analyses, and P-value below 0.05 was considered statistically significant. Normality of data distribution was tested with Shapiro-Wilks test. Student t-test, analysis of variance and χ^2^ post hoc test were performed to test the differences between groups. In case of normal distribution Mann-Whitney test was used. The correlation between eGFR and age subgroups was analyzed using Pearson correlation. Differences between three or more groups were analyzed in the case of normally distributed variables with ANOVA analysis of variance and Tukey-Kramer *post hoc* test.

## Results

A total of 1573 participants were enrolled, 1229 from EN villages and 344 from non-EN villages. In the group of endemic villages, 33 farmers were classified as having EN (2.7%), 86 as suspect of having EN (6.9%), 268 as those at risk (22%) and 842 were completely unrelated to EN – so called “others” (68%). We failed to find difference in gender prevalence within EN- and control villages. The average age of all participants was 52 years (range 18 - 90), with statistically significant difference in age between males and females in EN villages. In terms of body weight and SCr, there were statistically significant differences between males and females regardless of their place of recruitment, but also within EN- and control villages. No statistically significant differences regarding diabetes and hypertension were found between subjects from EN and control villages (Table 2). Prevalence of CKD in the Croatian rural area using CKD-EPI equation was very high (19%). Also, CKD prevalence was higher in females than in males (21% *vs.* 18%, P < 0.001) and significantly higher in EN then in non-EN villages in both sexes (P < 0.001) (Table 3). No differences were observed in the prevalence of CKD in hypertension and diabetes subgroups of participants between EN and non-EN villages (P = 0.704 and P = 0.945, respectively). The prevalence of CKD rose with patient’s age regardless of used equation. In EN diseased, CKD prevalence was the highest (88%).

**Table 2:** Basic participant characteristics in EN and non-EN villages

	**EN-villages**			**P**	**non EN-villages**			**P**
**All** **(N = 1229)**	**Females** **(N = 774)**	**Males** **(N = 455)**	**All** **(N = 344)**	**Females** **(N = 209)**	**Males** **(N = 135)**
Age (years)	51 (19 - 88)	52 (19 - 87)	50 (19 - 88)	0.032	53 (19 - 90)	53 (20 - 90)	54 (18 - 85)	0.420
Weight (kg)	78 ± 17	74 ± 16	85 ± 16	< 0.001	80 ± 16	76 ± 16	85 ± 14	< 0.001
Creatinine, serum (µmol/L)	93 ± 59	88 ± 60	102 ± 55	< 0.001	86 ± 51	79 ± 64	93 ± 12	< 0.001
Diabetes mellitus (%)	8.7	9.7	7.0	0.111	9.0	8.6	9.6	0.748
Hypertension (%)	37.0	37.0	37.0	0.935	38.0	37.0	40.0	0.635
EN - endemic nephropathy. Age is presented as median and range (min-max). Weight and creatinine concentrations are presented as mean ± standard deviation. Diabetes mellitus and hypertension subgroups were presented as proportions (%). P < 0.05 was considered statistically significant.								

**Table 3:** CKD prevalence and distribution of CKD stages in EN and non-EN villages

	**eGRF stage (%)**						**CKD prevalence (%)**
**G1**	**G2**	**G3a**	**G3b**	**G4**	**G5**
EN-villages (N = 1229)	35.0	51.0	7.7	4.1	1.5	0.8	14
non EN-villages (N = 344)	41.0	53.0	4.7	0.6	0.0	0.3	5.5
Data are shown as percentages for each eGFR stage. CKD prevalence was assessed using the eGFR calculated with CKD-EPI equation. eGFR - estimated glomerular filtration rate. CKD - chronic kidney disease. EN - endemic nephropathy.							

There was no statistically significant difference of eGFR between age groups in EN and non-EN villages. Compared according to gender, significantly higher eGFR were observed in males than females. This difference was more pronounced in subjects from EN villages (Table 4). The lowest eGFR value was expected in diseased (eGFR in all subgroups are presented in Table 5). There was no statistically significant difference between subjects with or without diabetes in eGFR. Between subjects with or without hypertension, no statistically significant difference was found in eGFR, with lower values in hypertensive subjects.

**Table 4:** eGFR according to CKD-EPI equation in EN and non-EN villages

	**EN-villages**			**P**	**Non EN-villages**			**P**
**All** **(N = 1229)**	**Females** **(N = 774)**	**Males** **(N = 455)**	**All** **(N = 344)**	**Females** **(N = 209)**	**Males** **(N = 135)**
eGFR (mL/min/1.73m^2^)	81 (80 - 82)	78 (77 - 80)	85 (83 - 87)	< 0.001	85 (84 - 87)	85 (83 - 87)	86 (83 - 89)	< 0.001
Data are shown as mean (95% confidence interval). P < 0.05 is considered statisticall significant. eGFR - estimated glomerular filtration rate. EN - endemic nephropathy.								

**Table 5:** eGFR in EN and control subgroups according to the CKD-EPI equation

**Subgroup**	**eGFR (mL/min/1.73m^2^)**
diseased (N = 33)	32 ± 27
suspected (N = 86)	64 ± 22
at risk (N = 268)	86 ± 17
others (N = 842)	83 ± 19
non-EN subjects (N = 344)	85 ± 17
Data shown as mean ± standard deviation. eGFR - estimated glomerular filtration rate. EN - endemic nephropathy.	

## Discussion

Diagnosis and staging of CKD depends on the correct calculation of GFR. We tested differences in eGFR among subjects from EN and non-EN areas by applying serum creatinine values to recommended CKD-EPI predictive equation.

CKD-EPI equation differentiated healthy from diseased subjects well. Participants from EN villages had higher prevalence of CKD than participants from non-EN villages. There are no new cases of EN today, and participants who were classified into diseased subgroup thirty years ago, today have more CKD_3-5_ stages than others in EN villages. No statistically significant difference was observed in the eGFR between patients with or without diabetes, although CKD-EPI equation underestimated their GFR (rather consistently with literature data) (*19*). In the hypertension group, significantly more G3a, G3b, G4 and G5 CKD stages were observed (20% *vs.* 7.7% in the non hypertension group). High prevalence of CKD_3-5_ stages in that subgroup could be a result of subjects lifestyle – high prevalence of obesity and hypertension, poor control of salt intake and untreated hypertension.

We are aware that this study has several limitations: recommended methods for serum creatinine measurements were not used in the study (enzymatic method has been applied in the Department of Laboratory Diagnostics, University Hospital Center Zagreb since 2014 and study is based on data collected between 2008 and 2010) and creatinine was determined only once so we do not know if there was a clinically significant difference in re-determining (based on the analytical and biological variation of serum creatinine the clinically significant difference for eGFR is about 14%). Also, eGFR in our study group, were not compared with the GFR measured using the gold standard with an ideal filtration marker.

However, the value of our study is that we found differences in the CKD prevalence and the frequency of certain stages of CKD in EN and non-EN villages. The prevalence of EN diseased (2.7%) and suspected (6.9%) patients is in accordance with the previously described incidence decline trend. This percentage of diseased and suspected patients explain the difference in CKD prevalence and it is an important indicator that in the coming years, regardless of if there will be new cases of EN, the number of new patients to start with renal replacement therapy will be higher in EN villages than in control. These results confirm that the assessment of kidney function with predictive equations proved to be a good marker in differentiating the study subgroups in the EN areas and it should therefore remain the diagnostic criteria for EN, and that the optimal equation for GFR estimation is CKD-EPI equation.
